# Supplementary material for: Pumpkin Oil and Its Effect on the Quality of Naples-Style Salami Produced from Buffalo Meat
Source: Foods. 2025 Mar 20;14(6):1077. doi: 10.3390/foods14061077 (PMC11942082; doi:10.3390/foods14061077)
Supplement: Supplementary file 1 [file foods-14-01077-s001.zip › foods-3529332-supplementary.pdf]

Table S1: Sensory analysis form

**Judge:**	**Sample:**								
**Smell**									
Intensity	1	2	3	4	5	6	7	8	9
	Meat family								
	Fresh meat			Sour meat			Fat		
	Aged meat				Other____________________				
	Animal Family								
	Stable		Gut						
Dominant Descriptor	Leather		Other __________________						
	Spicy Family								
	Green pepper		Garlic		Cinnamon			Red pepper	
	Nutmeg				Other____________________				
	Other								
	Acetic		Oxidized			Mold			
	Rancid		Ammonia			Other ________			
**Color**									
Intensity	1	2	3	4	5	6	7	8	9
	Pink								Intense red
Uniformity	1	2	3	4	5	6	7	8	9
	Not uniform								Uniform
**STRUCTURE**	**Visual perception**								
Compactness	1	2	3	4	5	6	7	8	9
Elasticity	1	2	3	4	5	6	7	8	9
Crushing	1	2	3	4	5	6	7	8	9
**Aroma**									
Intensity	1	2	3	4	5	6	7	8	9
	Meat family								
	Fresh meat			Sour meat			Fat		
	Aged meat				Other____________________				
	Animal Family								
	Stable		Casing						
Dominant Descriptor	Leather		Other ______________________________						
	Spicy Family								
	Pepper		Garlic		Cinnamon			Bell pepper	
	Nutmeg			Other______________________________					
	Off-Flavor								
	Acetic		Oxidized			Mold			
	Rancid		Ammonia			Other __________________			
**Taste**									
Salty	1	2	3	4	5	6	7	8	9
Acidic	1	2	3	4	5	6	7	8	9
Bitter	1	2	3	4	5	6	7	8	9
**TRIGEMINAL SENSATION**									
Spicy	1	2	3	4	5	6	7	8	9
**STRUCTURAL ATTRIBUTES**									
Hardness	1	2	3	4	5	6	7	8	9
Moisture	1	2	3	4	5	6	7	8	9
Chewability	1	2	3	4	5	6	7	8	9

Document S1

Consent Sensory Evaluations

Dear Sensory Panelist,

People 18 years of age and older are invited to participate in a research study to evaluate sensorial attributes of Napoli-salami style produced with buffalo meat enriched with pumpkin seed oil. This project is being conducted by the Department of Agricultural, Environmental and Food Sciences at the University of Molise.

PURPOSE OF THE STUDY: The sensory analyses are conducted by trained volunteers and are part of a larger study to identify user-friendly solutions based on the use pumpkin seed oil to improve the microbiological safety and consumer acceptability of Napoli-salami style produced with buffalo meat.

DETAILS:

- Dates: Each volunteer will participate in 10 training sessions and then perform a triple sensory analysis on four batches of Napoli-salami style produced with buffalo meat.
- Time Commitment: Each volunteer panelist participates in 10 training sessions and 3 sensory analysis sessions. Each session's duration is 60 minutes.
- Information Location: Sensory analyses are conducted at the Department's facilities

EXPLANATION OF PROCEDURES: Panelist conducts a threefold sensory analysis on the bar using a 9-point hedonic scale.

VOLUNTARITY: participation is voluntary, and the panelist may withdraw at any time without penalty.

Your help will be greatly appreciated in making this study meaningful.

PRIVACY AND CONFIDENTIALITY: All information obtained during the testing procedures will remain confidential and will not be visible to other panelists or people not associated with this study. The identity of the panelist will not be revealed in the results of the experiment. Only comparisons will be made and reported in summary form. The data will only be accessible to the researchers of the study and may only be used for the purpose of scientific publications in journals intended for the scientific community.

POTENTIAL BENEFITS AND RISKS: Participation in the study does not result in any direct benefits but does produce useful new knowledge in the field of food security.

CONTACT INFORMATION: If you have any questions about this project, please contact via email: tremonte@unimol.it or silvia.lombardi@unimol.it.

Documentation of Informed Consent:

You are freely making the decision to be in this research study. By signing this form, you indicate that:

1. You are 18 years of age or older,
2. you have read and understood this consent form,
3. you have had your questions answered,
4. you have decided to be in the study.

You will be given a copy of this consent form to keep.

____________________________________ ________________

Your Signature Date

Table S2 T-BARS values expressed as Malondialdehyde behavior (μg MDA/g sample) in samples from batches G20, conventional fermented sausages prepared with glucose 0,2%; batch G20P prepared with glucose 0,2% and pumpkin oil; batch G0, prepared without glucose 0,2%; batch G20P prepared with glucose 0,2% and pumpkin oil

Batches	0 days	49 days
**G20**	0.16 ± 0.02 [a]	0.78 ± 0.03 [a]
**G20P**	0.12 ± 0.03 [a]	0.38 ± 0.04 [b]
**G0**	0.17 ± 0.03 [a]	0.42 ± 0.05 [b]
**G0P**	0.14 ± 0.02 [a]	0.26± 0.02 [c]

The averages within the same column with different letters are significantly different ($P < 0.05$) based on the statistic ANOVA test

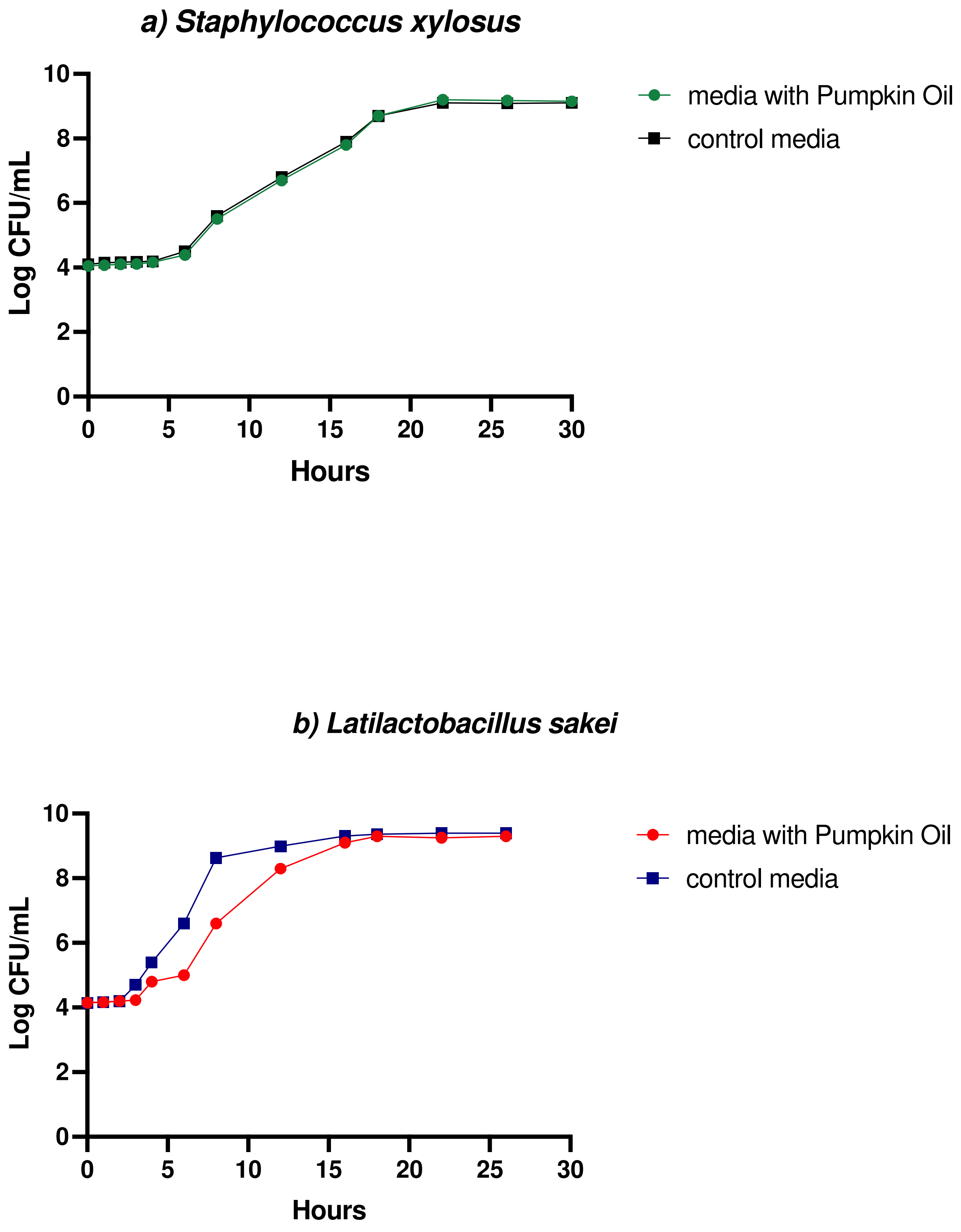

Figure S1. Trends of *S. xylosus* MVS9 and *Lt. sakei* 152 strains in a model food substrate with (media whit Pumpkin Oil) or without addition (control media) of pumpkin seed oil.
